# Effects of Tobacco Usage and Antiretroviral Therapy on Biomarkers of Systemic Immune Activation in HIV-Infected Participants

**DOI:** 10.1155/2018/8357109

**Published:** 2018-12-09

**Authors:** Helen C. Steel, W. D. Francois Venter, Annette J. Theron, Ronald Anderson, Charles Feldman, Luyanda Kwofie, Tanita Cronjé, Natasha Arullapan, Theresa M. Rossouw

**Affiliations:** ^1^Department of Immunology, University of Pretoria, South Africa; ^2^Institute for Cellular and Molecular Medicine, University of Pretoria, South Africa; ^3^Wits Reproductive Health and HIV Institute, University of the Witwatersrand, South Africa; ^4^Division of Pulmonology, Department of Internal Medicine, Charlotte Maxeke Johannesburg Academic Hospital and Faculty of Health Sciences, University of the Witwatersrand, South Africa; ^5^Tshwane Academic Division of the National Health Laboratory Service, Pretoria, South Africa; ^6^Department of Statistics, University of Pretoria, South Africa

## Abstract

Like HIV infection, smoking, which is common among HIV-infected persons, is associated with chronic, systemic inflammation. However, the possible augmentative effects of HIV infection and smoking and other types of tobacco usage on indices of systemic inflammation and the impact of combination antiretroviral therapy (cART) thereon remain largely unexplored and represent the focus of the current study. Of the total number of HIV-infected persons recruited to the study (*n* = 199), 100 were categorised as pre-cART and 99 as virally suppressed (HIV viral load < 40 copies/mL). According to serum cotinine levels, 144 and 55 participants were categorised as nonusers and users of tobacco, respectively. In addition to cytokines (IL-6, IL-8, and TNF-*α*) and chemokines (IP-10, MIG, IL-8, MCP-1, and RANTES), other biomarkers of systemic inflammation included C-reactive protein (CRP), *β*2-microglobulin, and those of neutrophil activation [ICAM-1, L-selectin, matrix metalloproteinase-9 (MMP-9)], microbial translocation (soluble CD14, LPS-binding protein), and oxidative stress (cyclophilin A, surfactant D). These were measured using multiplex bead array, ELISA, and immunonephelometric procedures. Viral suppression was associated with significant decreases in the levels of most of the biomarkers tested (*P* < 0.0037-0.0008), with the exceptions of CRP, cyclophilin A, and MMP-9. With respect to tobacco usage, irrespective of cART status, circulating levels of *β*2-microglobulin, cyclophilin A, and RANTES were significantly elevated (*P* < 0.042-0.012) in users vs nonusers. Additional analysis of the groups of tobacco users and nonusers according to cART status revealed high levels of RANTES in pre-cART/tobacco users relative to the three other subgroups (*P* < 0.004-0.0001), while more modest increases in cyclophilin A and MMP-9 (*P* < 0.019-0.027) were observed in comparison with the cART/tobacco user subgroup. Notwithstanding the efficacy of cART in attenuating HIV-associated, chronic systemic inflammation, the current study has identified RANTES as being significantly and seemingly selectively increased in those with active HIV infection who use tobacco, a mechanism which may underpin augmentative proinflammatory activity.

## 1. Introduction

According to UNAIDS, there were approximately 36.7 million people worldwide living with human immunodeficiency virus (HIV) in 2016. Of these, 19.5 million people were accessing combination antiretroviral therapy (cART) globally [1]. With the introduction of cART, the prevalence of AIDS-related mortality has declined significantly, with cART successfully suppressing viral replication to undetectable levels in plasma and increasing circulating CD4^+^ T cell numbers; however, it is unable to normalize immune activation [2]. Persistent immune activation and inflammation associated with HIV infection accelerate the process of immunosenescence [3], thereby potentially placing HIV-infected individuals at higher risk of developing infections and non-AIDS-defining diseases such as cancer (especially lung, head and neck, hepatocellular, Hodgkin's lymphoma, cervix, and anus) [4], cardiovascular diseases [5], renal [6] and liver [7] diseases, and neurocognitive impairment [8]. Most of these diseases have also been associated with concomitant tobacco use [9].

The prevalence of cigarette smoking among European and North American adults with HIV infection is higher (40-70%) [9, 10], compared to the general population (15-20%) [11], and these individuals are also less likely to stop smoking [12, 13]. Helleberg et al. reported that HIV-related and non-HIV-related disorders, as well as mortality, are significantly increased in HIV-infected individuals who also smoke cigarettes and that the increased mortality is associated with smoking rather than HIV-related factors [13]. In addition, smoking has been well documented to be a risk factor for respiratory complications associated with HIV infection such as tuberculosis and chronic obstructive pulmonary disease (COPD), as well as cryptococcosis, *Pneumocystis jirovecii*, and bacterial pneumonia [14–16]. HIV-1-infected smokers have also been shown to have decreased immune responses, poorer responses to cART, and a greater risk of biological rebound, compared to HIV-1-infected nonsmokers [17]. In this context, an increased level of hepatic CYP2B6 in smokers could lead to an altered metabolism of its substrates, thereby increasing the possibility of drug-drug interactions between nicotine and antiretroviral drugs (particularly protease inhibitors and nonnucleoside reverse transcriptase inhibitors) all of which are metabolised by cytochrome P450 enzymes [18, 19]. Subtherapeutic plasma levels of antiretrovirals, as well as overproduction of reactive oxygen species (ROS), resulting from these interactions may lead to enhanced viral replication [18, 20].

Moreover, cigarette smoking affects both innate and adaptive (cell-mediated and humoral) immune responses (reviewed by [21–26]) and has been associated with both release and inhibition of proinflammatory and anti-inflammatory mediators. Cells of the adaptive immune system affected by smoking include T helper cells (Th1/Th2/Th17), CD4^+^CD25^+^ regulatory T cells, CD8^+^ T cells, B cells, and memory T/B lymphocytes, while cells of the innate immune system affected by smoking include dendritic cells, macrophages, and natural killer cells [24].

With respect to the proinflammatory activity, the nuclear factor kappa-light-chain-enhancer of activated B cell (NF-*κ*B) signalling pathway is well recognized as one of the main mechanisms underpinning smoking-induced activation of inflammatory responses triggered through activation of the inhibitor of NF-*κ*B kinase. Translocation of active NF-*κ*B to the nucleus then occurs, followed by the induction of a number of genes involved in immune regulation and inflammation [27–30]. Proinflammatory cytokines shown to be upregulated after exposure to cigarette smoke condensate include tumour necrosis factor-*α* (TNF-*α*), interleukin- (IL-) 1, IL-6, IL-8, granulocyte-macrophage colony-stimulating factor (GM-CSF), and monocyte chemotactic protein-1 (MCP-1) [28, 30].

The association between HIV, smoking, and increased upregulation and release of potent cytokines and chemokines has been investigated in a number of studies [21, 27, 31], and, notably, it has been suggested that cytokine dysregulation may eventually contribute to HIV disease progression [31]. In addition, cytokines have been associated with the development of viral latency and the maintenance of latently infected CD4^+^ T cells during cART, with potentially important implications for viral persistence posing a barrier to HIV cure [32].

Importantly, however, the effects of tobacco usage on the systemic inflammatory profiles of HIV-infected persons are largely unexplored, as is the influence of cART. The primary focus of the current study is on the cumulative effects of active HIV infection and tobacco usage, as well as the impact of cART, on a profile of inflammatory biomarkers.

## 2. Methods

### 2.1. Study Population

Adult (≥18 years) HIV-infected participants attending the Antiretroviral Clinic at the Charlotte Maxeke Johannesburg Academic Hospital in Johannesburg, South Africa, were recruited by means of convenience sampling. Ethics approval for the study was obtained from the Faculty of Health Sciences Research Ethics Committee, University of Pretoria, Pretoria, South Africa (Ethics reference number 94/2013), and the Human Research Ethics Committee of the University of the Witwatersrand, Johannesburg, South Africa (Clearance Certificate Number M130383). All participants gave informed consent, and a questionnaire was completed containing patient demographic data, and information related to the use of tobacco products. Treatment information was retrieved from clinical records. CD4^+^ lymphocyte counts and HIV-RNA levels were measured by standard flow cytometric and PCR-based procedures, respectively, according to the South African National Health Laboratory Services guidelines. In addition, whole blood samples were collected in EDTA vacutainers and processed within 4 hours to separate the plasma component by centrifugation. The resultant plasma was stored at minus 80°C for up to 24 months.

The patients were classified in two groups, *viz.*, those who were cART-naïve (*n* = 100) and those who were virologically suppressed on their cART regimen [HIV-1 plasma viral load (VL) < 40 copies/mL] (*n* = 99).

### 2.2. Measurement of Circulating Biomarkers of Immune Activation

The selection of biomarkers used in this study was based on previous findings with the inclusion of several additional markers [33, 34].

#### 2.2.1. Cytokines

IL-6, IL-8 (CXCL8), IL-17, monokine induced by gamma interferon (MIG, CXCL9), IFN-*γ*, monocyte chemoattractant protein-1 (MCP-1, CCL2), interferon gamma-induced protein-10 (IP-10, CXCL10), TNF-*α*, and regulated on activation, normal T cell expressed and secreted (RANTES) were quantified using the Bio-Plex suspension array platform together with Bio-Plex Pro™ assay kits (Bio-Rad Laboratories Inc., Hercules, CA, USA) according to the manufacturer's instructions.

#### 2.2.2. Neutrophil Activation Markers

Biomarkers of neutrophil activation included L-selectin (CD62L), intercellular adhesion molecule-1 (ICAM-1, CD54), and matrix metalloproteinase-9 (MMP-9, gelatinase B) (R&D Systems, Minneapolis, MN, USA), as well as IL-8 (as mentioned above), the levels of which were determined by conventional ELISA kits.

#### 2.2.3. Oxidative Stress Marker [35]

Cyclophilin A (Elabscience, Wuhan, China) levels were measured by standard ELISA.

#### 2.2.4. Microbial Translocation and Monocyte Activation

Soluble CD14 (sCD14) (biomarker of monocyte activation) (Abcam, Cambridge MN, USA) and surfactant protein D (biomarkers of the integrity of the lung epithelium) (Hycult Biotech, Plymouth Meeting, PA, USA) and lipopolysaccharide-binding protein (LBP) (R&D Systems, Minneapolis, MN, USA) levels were determined using standard ELISA methods.

#### 2.2.5. Infection and Inflammation

C-reactive protein (hsCRP) and *β*2-microglobulin levels were assayed by using high-sensitivity nephelometry (Siemens Healthcare Diagnostics, South Africa).

### 2.3. Smoking Status

Cotinine, as an objective measure of tobacco use, was measured using an ELISA procedure (Calbiotech, Spring Valley, CA, USA), with levels above 15 ng/mL taken as being positive for active smoking [36]. Because of the prevalence of usage of snuff among older, black South African females in particular, with reported frequencies of around 43%-48% [37, 38], together with the inability of measurement of serum cotinine to distinguish between active smoking and usage of inhaled snuff, we have chosen to use the term “tobacco users” as opposed to “smokers” in the current report.

### 2.4. Statistical Analysis

Data followed a nonnormal distribution, and distribution-free statistics were therefore employed. Descriptive results are given as proportions and frequencies, as well as medians and interquartile ranges (IQR). Tests of association were performed by chi-square or Fisher's exact tests as appropriate, or by the Kruskal-Wallis test, used for comparing two or more independent samples.

## 3. Results

A total of 199 participants were recruited for the study of whom 100 were cART-naïve and 99 were virologically suppressed on cART. Participants had a median age of 33 years [interquartile range (IQR) 30–39 years], all but 3 were Black African, and the majority (59.3%) were women. One hundred and thirty-eight participants (69.4%) admitted to have ever smoked, and 55 (27.6%) were current tobacco users as determined by cotinine levels. Fifty-three participants used snuff and 55 smoked cigarettes and had been doing so for a median duration of 5 (IQR 4.5–15) and 19 (IQR 17–27) years, respectively, with 38 using both. Smokers smoked on average 5 (IQR 3–7) cigarettes per day. Males were significantly more likely to use tobacco products than females were (37% versus 21.2%; *P* = 0.014). All cART-treated participants were on standard first-line treatment consisting of tenofovir disoproxil fumarate, emtricitabine, and efavirenz which had been started a median of 14 (IQR 14–19) months earlier, and all participants had an undetectable VL at the time of the study.

Table 1 shows the demographic, clinical, and biomarker results of participants according to cART status with comparisons between the groups. Participants did not differ according to age or gender, but treatment-naïve participants had significantly lower CD4 counts and higher levels of all biomarkers tested, except for MMP-9, which was lower, and hsCRP and cyclophilin A, which were comparable to values observed in virally suppressed participants. Apart from gender, which is shown as frequency and percentage, all results are given as median and IQR throughout.

**Figure 1:**
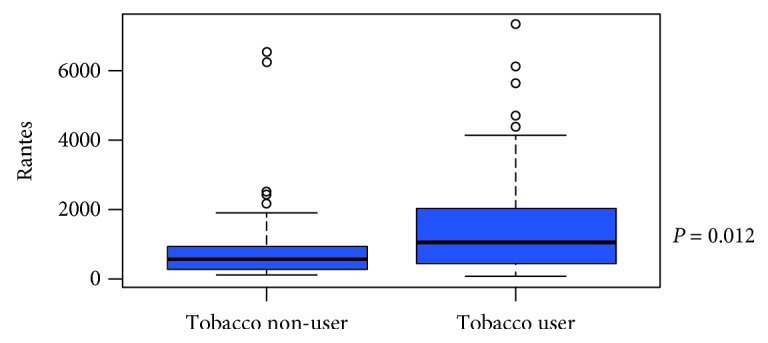
Boxplot of RANTES according to tobacco usage.

**Figure 2:**
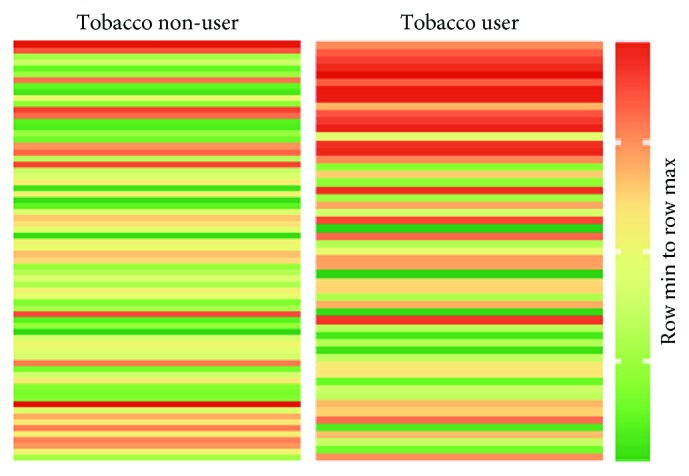
Heat map of RANTES according to tobacco usage.

Table 2 shows the demographic, clinical, and biomarker results of participants according to tobacco usage, as determined by cotinine level, with comparisons between the groups. Tobacco users were more likely to be male and had comparable age, CD4 counts, and VL than nonusers, but significantly higher levels of RANTES (Figures 1 and 2), *β*2-microglobulin, and cyclophilin A.

Figure 1 displays the distribution of the RANTES values, illustrating that the tobacco users have significantly higher levels of RANTES than nonusers. Additionally, Figure 2 provides an immediate visual summary of the information showcasing those high values found in the tobacco users group.

Table 3 shows the demographic, clinical, and biomarker results of participants according to combined cART status and tobacco use. No differences were found in hsCRP between any of the groups, and cyclophilin A only differed between treatment-naïve tobacco users and nonusers, with levels significantly higher in the former group. RANTES was strikingly higher in the treatment-naïve tobacco users when compared with all the other groups. Treatment-naïve and virologically suppressed tobacco users differed significantly in all biomarkers (all higher in the former) apart from MMP-9, sCD14, and surfactant-D. A similar pattern was observed in treatment-naïve and virologically suppressed tobacco nonusers, except that no difference was observed for RANTES.

When treatment-naïve tobacco users were compared with nonusers, differences were only found in RANTES, MMP-9, and cyclophilin A, whereas no differences were found in the virally suppressed group. Finally, treatment-naïve tobacco users had significantly higher levels of all biomarkers, except for MMP-9, than nonusers on cART, while tobacco users on cART and nonusers not on cART had variable patterns with MMP-9 higher in the former group and MIG, IL-6, IL-8, IP-10, MCP-1, TNF-*α*, L-selectin, ICAM-1, LBP, and *β*2-microglobulin higher in the latter group.

## 4. Discussion

Our findings add substantially to the limited literature pertaining to the immunomodulatory effects of cART *per se*, especially in African populations. As described previously by us and others, most biomarkers of systemic inflammation in adults infected with HIV decrease progressively and significantly following the implementation of virally suppressive cART irrespective of viral subtype B or C [33, 39–42]. This scenario is strongly supported by the findings of the current study, specifically with respect to the test cytokines (IL-6, TNF-*α*), chemokines (IP-10, MIG, MCP-1, and RANTES), *β*2-microglobulin, and two of the three biomarkers of microbial translocation (sCD14, LBP). These findings underscore the apparent utility of measurement of IP-10, in particular, as an objective indicator of responsiveness to cART [33, 39, 42, 43], a contention which is in keeping with the role of this chemokine in the pathogenesis of HIV infection [43–46].

Although previous studies have described elevations in the levels of individual biomarkers associated with neutrophilic inflammation, specifically ICAM-1 [47], L-selectin [48], and IL-8 [49], characterization of the effects of cART on a profile of biomarkers associated with systemic activation of these cells has not, to our knowledge, been described previously. In this context, our observations that administration of cART is associated with significant decreases in the concentrations of ICAM-1, L-selectin, and IL-8 is consistent with systemic activation of these cells [50, 51] and a beneficial, albeit secondary, anti-inflammatory effect of therapy. This interpretation is supported by the observation that virally suppressive cART was also associated with a significant decrease in serum surfactant D, a biomarker of oxidative stress [52].

According to the findings of two studies, South African HIV-infected participants, particularly males, like their European and North American counterparts, have high rates of cigarette smoking with frequencies of 52–71% recorded in males and 13–28% in females [34, 37, 53], with somewhat lower rates recorded in another study which was undertaken at the same HIV clinic as the current study [54].

With respect to characterization of the effects of tobacco usage on systemic biomarker profiles, we first categorised the entire cohort of HIV-infected participants into two groups according to low or high serum cotinine levels (nonusers and predominantly male tobacco users accounting for 72.4% and 27.6% of participants in each group, respectively), irrespective of cART. Although tobacco usage was associated with increases in almost all of the test protein biomarkers, consistent with a more systemic inflammatory phenotype, only three of these attained statistical significance, *viz.*, the chemokine, RANTES, a key mediator of T lymphocyte recruitment and activation, and *β*2-microglobulin and cyclophilin A. The latter biomarker is released from various cell types, including vascular smooth muscle cells, inflammatory cells, and platelets during systemic oxidative stress, including that caused by smoking [55].

These findings are relevant in the context of a recent study which reported that mild-to-moderate smoking is associated with significant increases in VL, as well as with increases in the systemic concentrations of RANTES and biomarkers of oxidative stress, while no alterations were detected in the plasma levels of several other proinflammatory cytokines/chemokines, specifically IL-1*β*, IL-6, TNF-*α*, IL-8, and MCP-1 [56]. In this context, it is noteworthy that RANTES has been implicated in microbial translocation [57], a process known to drive systemic inflammation, as well as in the pathogenesis of COPD [58, 59] and coronary artery disease (CAD) [60–62], conditions in which smoking is well recognized as being a key risk factor, particularly in HIV-infected individuals who smoke [16, 23, 63]. In addition, elevated plasma levels of cyclophilin A have recently been described in both CAD [55, 64] and COPD [65].

Much less is known about possible relationships between circulating levels of *β*2-microglobulin and tobacco usage, particularly in the setting of HIV infection. A few studies have, however, reported on elevated levels of this HLA class 1-derived biomarker in the serum and urine of HIV-uninfected smokers. These have been attributed to the cytotoxic activities of smoke-derived toxicants, particularly cadmium, predisposing for possible development of nephropathy [66], malignancy [67], and ischemic stroke [68]. In those infected with HIV who smoke, exposure to smoke-derived toxicants may also contribute to T cell dysfunction and death [56].

Mostly in keeping with the aforementioned findings, further subdivision of tobacco users/nonusers according to cART status revealed significantly elevated levels of RANTES in particular, as well as cyclophilin A and MMP-9, but not *β*2-microglobulin, in tobacco users not on cART (hence with high VL) relative to nonusers not on cART. Interestingly, systemic levels of RANTES were significantly higher in the no cART/user group than in any of the other subgroups (no cART/nonuser, cART/user, and cART/nonuser), as also reported in another study, albeit in a much smaller group of HIV-infected patients of unknown treatment status [69]. Following initiation of cART, levels of RANTES decreased strikingly, attaining levels comparable to those of nonsmokers. Aside from direct antiviral activity, these notable effects of cART may also be attributed to attenuation of microbial translocation, a contention which is consistent with the decline in post-therapy levels of LBP. These findings suggest that RANTES may represent a selective biomarker of the harmful, augmentative, proinflammatory interactions of active HIV infection and smoking, while also underscoring the anti-inflammatory benefit of cART. It is however, noteworthy, that conflicting data exist regarding the role of RANTES in HIV infectivity and disease progression [70].

With respect to MMP-9, this proteolytic enzyme, which is mainly produced by activated neutrophils, as well as by monocytes/macrophages, is elevated in the blood and lungs of smokers and is intimately involved in the pathogenesis of COPD [71–74]. The absence of a significant difference in *β*2-microglobulin in the tobacco-user versus nonuser groups on cART may reflect the dominant influence of active viral replication on levels of this biomarker.

Limitations of the current study include lack of VL data in the pre-cART group, small numbers of HIV-infected tobacco users when subdivided into cART subgroups, and lack of distinction between active smoking and usage of smokeless tobacco products. Irrespective of these limitations, this study has clearly reinforced the benefit of cART per se in attenuating HIV-associated, harmful, chronic, systemic inflammation, including neutrophilic inflammation, as well as the utility of IP-10 as a biomarker of the efficacy of cART. More importantly, however, the finding, albeit preliminary, that levels of RANTES are considerably and possibly selectively increased in individuals with active HIV infection who use tobacco implies a major role for this chemokine in the pathogenesis of HIV/smoking-related immune dysfunction and organ damage. These findings again highlight the damaging interactions between active HIV infection and tobacco use, underscoring the need to prioritise effective smoking cessation strategies in this population.

## Figures and Tables

**Table 1:** Demographic, clinical, and biomarker results of pre-cART and virally suppressed participants.

	Pre-cART *n* = 100	Virally suppressed *n* = 99	*P* value
Age (years)	33 (29–37)	34 (31–41)	0.102
Gender	Female = 64 (64%)	Female = 54 (54.5%)	0.175
CD4 (cells/*μ*L)	209 (115–305)	348 (240–464)	**0.0001**
Current smoker	32 (32%)	23 (23.2%)	0.167
Cotinine (ng/mL)	0 (0–87.3)	0 (0–9.5)	0.139
MIG (pg/mL)	2610.61 (1353.1–4350.02)	949.26 (694.21–1371.34)	**0.0001**
IL-6 (pg/mL)	4.51 (2.69–8.14)	2.26 (1.41–3.12)	**0.0001**
IL-8 (pg/mL)	22.0 (10.76–45.36)	8.2 (5.64–12.47)	**0.0001**
IP-10 (pg/mL)	1896.98 (963.88–2990.94)	723.13 (567.82–977.66)	**0.0001**
MCP-1 (pg/mL)	23.21 (13.39–41.81)	5.13 (0–21.66)	**0.0001**
TNF-*α* (pg/mL)	5.04 (0–14.14)	0 (0–2.44)	**0.0001**
RANTES (pg/mL)	1203.68 (326.58–2444.85)	563.22 (313.21–935.84)	**0.0037**
MMP-9 (ng/mL)	32.08 (18.77–56.51)	39.17 (28.54–53.7)	**0.038**
L-selectin (ng/mL)	1189.85 (1029.95–1418.19)	924.86 (781.39–1108.35)	**0.0001**
ICAM-1 (ng/mL)	351.51 (187.97–530.18)	185.42 (107.04–240.38)	**0.0001**
sCD14 (ng/mL)	11750.64 (8039.79–17467.5)	9029.48 (6836.25–12,373)	**0.0003**
Surfactant-D (ng/mL)	288.17 (169.5–448.52)	210.95 (123.73–340.87)	**0.003**
LBP (ng/mL)	2734.865 (1740.25–4286.065)	1151.46 (956.65–1441.26)	**0.0001**
*β*2-microglobulin (*μ*g/mL)	2.98 (2.12–3.88)	1.7 (1.5–2.09)	**0.0001**
hsCRP (*μ*g/mL)	3.84 (1.08–8.65)	3.03 (1.02–6.28)	0.194
Cyclophilin A (ng/mL)	33.79 (15.56–58.85)	33.01 (19.5–51.05)	0.794

**Table 2:** Demographic, clinical, and biomarker results according to tobacco usage.

	Tobacco nonuser *n* = 144	Tobacco user *n* = 55	*P* value
Age (years)	33 (29.5–39)	34 (30–39)	0.827
Gender	Female = 93 (64.6%)	Female = 25 (45.5%)	**0.014**
BMI (kg/m^2^) (*n* = 77; *n* = 23)	26 (23–30)	23 (21–27)	0.084
CD4 (cells/*μ*L)	283 (181–366)	294.5 (184–334)	0.815
VL (copies/mL) (*n* = 76; *n* = 23)	40 (40–40)	40 (40–40)	1.000
Cotinine (ng/mL)	0 (0-0)	100 (84–100)	**0.0001**
MIG (pg/mL)	1202.27 (705.05–2381.51)	1586.55 (1118.17–3140.22)	0.070
IL-6 (pg/mL)	2.9 (1.73–5.36)	3.12 (2.26–6.11)	0.404
IL-8 (pg/mL)	11.19 (6.07–23.96)	12.47 (9.06–31.27)	0.296
IP-10 (pg/mL)	923.06 (618.67–1821.06)	1177.58 (699.38–2030.72)	0.249
MCP-1 (pg/mL)	13.91 (3.58–27.86)	21.66 (7.19–31.99)	0.114
TNF-*α* (pg/mL)	0.57 (0–5.69)	1.14 (0–10.24)	0.505
RANTES (pg/mL)	552.31 (279.88-934.91)	1059.49 (436.52-2043.34)	**0.012**
MMP-9 (ng/mL)	34.74 (22.36–53.7)	39.17 (26.77–67.08)	0.161
L-selectin (ng/mL)	1060.95 (881.17–1283.41)	1076.63 (809.64–1223.38)	0.543
ICAM-1 (ng/mL)	223.31 (133.7–351.51)	205.88 (130.82–427.59)	0.827
sCD14 (ng/mL)	10177.13 (6859.92–14065.46)	10696.59 (7720.14-14664.51)	0.516
Surfactant-D (ng/mL)	239.33 (138.28–365.35)	286.61 (184.47–370.3)	0.143
LBP (ng/mL)	1512.9 (1085.55–2803.38)	1941.39 (1168.96–3235.82)	0.305
*β*2-Microglobulin (*μ*g/mL)	1.99 (1.52–2.97)	2.33 (1.82–3.06)	**0.042**
hsCRP (*μ*g/mL)	3.56 (1.14–7.48)	2.71 (0.817–6.85)	0.349
Cyclophilin A (ng/mL)	29.19 (16.3–51.73)	39.44 (23.46–73.61)	**0.033**

**Table 3:** Demographic, clinical, and biomarker results according to treatment status and tobacco use.

	Tobacco user		Tobacco nonuser		*P* value					
	1 cART-*n* = 32	2 cART + *n* = 23	3 cART-*n* = 68	4 cART + *n* = 76	1 vs 2	1 vs 3	1 vs 4	2 vs 3	2 vs 4	3 vs 4
Age (years)	33.5 (28.5–37)	35 (31–41)	32 (29–37.5)	34 (30.5–40)	0.165	0.420	0.192	0.099	0.365	0.087
Gender	*F* = 19 (59.38%)	*F* = 6 (26.09%)	*F* = 45 (66.18%)	*F* = 48 (63.16%)	**0.007**	0.260	0.358	**0.0004**	**0.0008**	0.357
CD4 (cells/*μ*L)	211 (137.5–305)	344 (315–466)	209 (106–307)	351.5 (237.5–454.5)	**0.0001**	0.413	**0.0001**	**0.0001**	0.267	**0.0001**
Cotinine (ng/mL)	100 (100–100)	100 (67.3–100)	0 (0–0)	0 (0–0)	0.363	**0.0001**	**0.0001**	**0.0001**	**0.0001**	0.361
MIG (pg/mL)	2696.0 (1786.43–3460.14)	1118.17 (787.12–1383.5)	2501.3 (1110.43–5854.07)	905.33 (677.94–1328.77)	**0.0001**	0.143	**0.0001**	**0.0001**	0.334	**0.0001**
IL-6 (pg/mL)	3.55 (2.37–8.99)	2.26 (1.84–4.4)	5.04 (2.8–8.14)	1.84 (1.31–3.12)	**0.046**	0.132	**0.0001**	**0.002**	0.060	**0.0001**
IL-8 (pg/mL)	18.46 (9.91–47.13)	9.06 (7.35–17.6)	22.72 (10.76–41.09)	7.35 (4.78–12.47)	**0.013**	0.247	**0.0001**	**0.0008**	0.090	**0.0001**
IP-10 (pg/mL)	1947.09 (1330.02–2701.1)	695.91 (545.75–865.47)	1821.06 (833.45–4174.76)	751.84 (570.31-1008.06)	**0.0001**	0.114	**0.0001**	**0.0001**	0.236	**0.0001**
MCP-1 (pg/mL)	26.31 (16.49–41.29)	5.13 (0.99–23.72)	22.69 (11.84–41.81)	5.13 (0–20.62)	**0.0001**	0.193	**0.0001**	**0.0003**	0.337	**0.0001**
TNF-*α* (pg/mL)	7.64 (0–15.44)	0 (0–1.14)	4.39 (0–11.54)	0 (0–2.44)	**0.0001**	0.187	**0.0001**	**0.0001**	0.217	**0.0001**
RANTES (pg/mL)	1698.37 (560.79–3655.84)	488.02 (226.59–1114.67)	478.55 (214.02–1785.06)	564.24 (317.82–861.03)	**0.001**	**0.004**	**0.0002**	0.382	0.491	0.372
MMP-9 (ng/mL)	38.28 (19.66–73.02)	39.17 (33.85–50)	28.54 (18.77–50)	38.28 (28.54–54.64)	0.284	**0.049**	0.461	**0.017**	0.285	**0.012**
L-selectin (ng/mL)	1165.21 (932.31–1327.36)	924.86 (614.35–1092.43)	1189.85 (1045.45–1455.75)	932.22 (802.57–1108.35)	**0.0008**	0.163	**0.0005**	**0.0001**	0.233	**0.0001**
ICAM-1 (ng/mL)	360.41 (185.34–537.23)	153.38 (113.1–205.88)	351.51 (195.56–507.0)	190.6 (100.89–240.38)	**0.0006**	0.339	**0.0001**	**0.0001**	0.436	**0.0001**
sCD14 (ng/mL)	11285.24 (8922.59–15044.95)	9202.32 (7296.24–13,749)	12846.1 (7273.22–18604.36)	8993.65 (6678.74–12346.9)	0.112	0.385	**0.007**	0.051	0.222	**0.0003**
Surfactant-D (ng/mL)	325.17 (178.82–388.71)	273.49 (184.47–354.23)	272.63 (165.24–460.77)	186.31 (114.11–315.62)	0.290	0.433	**0.006**	0.3161	0.057	**0.0016**
LBP (ng/mL)	2844.95 (1943.9–4057.12)	1103.18 (828.71–1441.26)	2640.38 (1627.25–4494.89)	1158.02 (972.33–1443.51)	**0.0001**	0.311	**0.0001**	**0.0001**	0.369	**0.0001**
*β*2-Micro-globulin (*μ*g/mL)	2.97 (2.26–3.5)	1.88 (1.52–2.14)	2.98 (1.84–4.5)	1.69 (1.47–2.08)	**0.0001**	0.193	**0.0001**	**0.0001**	0.174	**0.0001**
hsCRP (*μ*g/mL)	2.25 (0.86–6.93)	3.15 (0.77–6.85)	4.34 (1.15–11.75)	2.96 (1.14–6.28)	0.443	0.105	0.482	0.101	0.418	0.061
Cyclophilin A (ng/mL)	40.01 (26.2–76.32)	36.7 (19.5–64.29)	24.7 (14.6–56.64)	30.96 (19.51–47.82)	0.437	**0.027**	0.074	0.063	0.136	0.258

## Data Availability

The data used to support the findings of this study are available from the corresponding author upon request.

## References

[1] UNAIDS Fact sheet – latest statistics on the status of the AIDS epidemic: Global HIV statistics. http://www.unaids.org/en/resources/fact-sheet.

[2] Cassol E., Malfeld S., Mahasha P. (2010). Persistent microbial translocation and immune activation in HIV-1-infected South Africans receiving combination antiretroviral therapy. *Journal of Infectious Diseases*.

[3] Sokoya T., Steel H. C., Nieuwoudt M., Rossouw T. M. (2017). HIV as a cause of immune activation and immunosenescence. *Mediators of Inflammation*.

[4] Wang C. J., Silverberg M. J., Abrams D. I. (2014). Non-AIDS-defining malignancies in the HIV-infected population. *Current Infectious Disease Reports*.

[5] Ballocca F., D’Ascenzo F., Gili S., Grosso Marra W., Gaita F. (2017). Cardiovascular disease in patients with HIV. *Trends in Cardiovascular Medicine*.

[6] Szczech L. A., Hoover D. R., Feldman J. G. (2004). Association between renal disease and outcomes among HIV-infected women receiving or not receiving antiretroviral therapy. *Clinical Infectious Diseases*.

[7] Weber R., Sabin C. A., Friis-Moller N. (2006). Liver-related deaths in persons infected with the human immunodeficiency virus: the D:A:D study. *Archives of Internal Medicine*.

[8] Carroll A., Brew B. (2017). HIV-associated neurocognitive disorders: recent advances in pathogenesis, biomarkers, and treatment. *F1000Research*.

[9] Lifson A. R., Neuhaus J., Arribas J. R., van den Berg-Wolf M., Labriola A. M., Read T. R. (2010). Smoking-related health risks among persons with HIV in the Strategies for Management of Antiretroviral Therapy clinical trial. *American Journal of Public Health*.

[10] Tesoriero J. M., Gieryic S. M., Carrascal A., Lavigne H. E. (2010). Smoking among HIV positive New Yorkers: prevalence, frequency, and opportunities for cessation. *AIDS and Behavior*.

[11] Jamal A., Phillips E., Gentzke A. S. (2018). Current cigarette smoking among adults — United States, 2016. *MMWR Morbidity and Mortality Weekly Report*.

[12] Mdodo R., Frazier E. L., Dube S. R. (2015). Cigarette smoking prevalence among adults with HIV compared with the general adult population in the United States: cross-sectional surveys. *Annals of Internal Medicine*.

[13] Helleberg M., Afzal S., Kronborg G. (2013). Mortality attributable to smoking among HIV-1-infected individuals: a nationwide, population-based cohort study. *Clinical Infectious Diseases*.

[14] van Zyl-Smit R. N., Brunet L., Pai M., Yew W. W. (2010). The convergence of the global smoking, COPD, tuberculosis, HIV, and respiratory infection epidemics. *Infectious Disease Clinics of North America*.

[15] Petrosillo N., Cicalini S. (2013). Smoking and HIV: time for a change?. *BMC Medicine*.

[16] Rossouw T. M., Anderson R., Feldman C. (2015). Impact of HIV infection and smoking on lung immunity and related disorders. *European Respiratory Journal*.

[17] Feldman J. G., Minkoff H., Schneider M. F. (2006). Association of cigarette smoking with HIV prognosis among women in the HAART era: a report from the women’s interagency HIV study. *American Journal of Public Health*.

[18] Ande A., McArthur C., Kumar A., Kumar S. (2013). Tobacco smoking effect on HIV-1 pathogenesis: role of cytochrome P450 isozymes. *Expert Opinion on Drug Metabolism & Toxicology*.

[19] Earla R., Ande A., McArthur C., Kumar A., Kumar S. (2014). Enhanced nicotine metabolism in HIV-1-positive smokers compared with HIV-negative smokers: simultaneous determination of nicotine and its four metabolites in their plasma using a simple and sensitive electrospray ionization liquid chromatography-tandem mass spectrometry technique. *Drug Metabolism and Disposition*.

[20] Pal D., Kwatra D., Minocha M., Paturi D. K., Budda B., Mitra A. K. (2011). Efflux transporters- and cytochrome P-450-mediated interactions between drugs of abuse and antiretrovirals. *Life Sciences*.

[21] Arnson Y., Shoenfeld Y., Amital H. (2010). Effects of tobacco smoke on immunity, inflammation and autoimmunity. *Journal of Autoimmunity*.

[22] Feldman C., Anderson R. (2013). Cigarette smoking and mechanisms of susceptibility to infections of the respiratory tract and other organ systems. *The Journal of Infection*.

[23] Calvo M., Laguno M., Martínez M., Martínez E. (2015). Effects of tobacco smoking on HIV-infected individuals. *AIDS Reviews*.

[24] Qiu F., Liang C. L., Liu H. (2017). Impacts of cigarette smoking on immune responsiveness: up and down or upside down?. *Oncotarget*.

[25] Nizri E., Irony-Tur-Sinai M., Lory O., Orr-Urtreger A., Lavi E., Brenner T. (2009). Activation of the cholinergic anti-inflammatory system by nicotine attenuates neuroinflammation via suppression of Th1 and Th17 responses. *The Journal of Immunology*.

[26] Strzelak A., Ratajczak A., Adamiec A., Feleszko W. (2018). Tobacco smoke induces and alters immune responses in the lung triggering inflammation, allergy, asthma and other lung diseases: a mechanistic review. *International Journal of Environmental Research and Public Health*.

[27] Goncalves R. B., Coletta R. D., Silvério K. G. (2011). Impact of smoking on inflammation: overview of molecular mechanisms. *Inflammation Research*.

[28] Yang S. R., Chida A. S., Bauter M. R. (2006). Cigarette smoke induces proinflammatory cytokine release by activation of NF-*κ*B and posttranslational modifications of histone deacetylase in macrophages. *American Journal of Physiology Lung Cellular and Molecular Physiology*.

[29] Oeckinghaus A., Ghosh S. (2009). The NF-*κ*B family of transcription factors and its regulation. *Cold Spring Harbor Perspectives in Biology*.

[30] Lerner L., Weiner D., Katz R., Reznick A. Z., Pollack S. (2009). Increased pro-inflammatory activity and impairment of human monocyte differentiation induced by in vitro exposure to cigarette smoke. *Journal of Physiology and Pharmacology*.

[31] Valiathan R., Miguez M. J., Patel B., Arheart K. L., Asthana D. (2014). Tobacco smoking increases immune activation and impairs T-cell function in HIV infected patients on antiretrovirals: a cross-sectional pilot study. *PLoS One*.

[32] Vandergeeten C., Fromentin R., Chomont N. (2012). The role of cytokines in the establishment, persistence and eradication of the HIV reservoir. *Cytokine & Growth Factor Reviews*.

[33] Malherbe G., Steel H. C., Cassol S. (2014). Circulating biomarkers of immune activation distinguish viral suppression from nonsuppression in HAART-treated patients with advanced HIV-1 subtype C infection. *Mediators of Inflammation*.

[34] Makhubele T. G., Steel H. C., Anderson R., van Dyk G., Theron A. J., Rossouw T. M. (2016). Systemic immune activation profiles of HIV-1 subtype C-infected children and their mothers. *Mediators of Inflammation*.

[35] Satoh K., Shimokawa H., Preedy V., Patel V. (2015). Cyclophilin A: novel biomarker for oxidative stress and cardiovascular diseases. *General Methods in Biomarker Research and their Applications. Biomarkers in Disease: Methods, Discoveries and Applications*.

[36] SNRT Subcommittee on Biochemical Verification (2002). Biochemical verification of tobacco use and cessation. *Nicotine & Tobacco Research*.

[37] Elf J. L., Variava E., Chon S. (2017). Prevalence and correlates of smoking among people living with HIV in South Africa. *Nicotine & Tobacco Research*.

[38] Govind N., Ally M. M., Tikly M., Anderson R., Hodkinson B., Meyer P. W. (2016). Pitfalls in the assessment of smoking status detected in a cohort of South African RA patients. *Rheumatology International*.

[39] Kamat A., Misra V., Cassol E. (2012). A plasma biomarker signature of immune activation in HIV patients on antiretroviral therapy. *PLoS One*.

[40] Wada N. I., Jacobson L. P., Margolick J. B. (2015). The effect of HAART-induced HIV suppression on circulating markers of inflammation and immune activation. *AIDS*.

[41] Maina E. K., Abana C. Z., Bukusi E. A., Sedegah M., Lartey M., Ampofo W. K. (2016). Plasma concentrations of transforming growth factor beta 1 in non-progressive HIV-1 infection correlates with markers of disease progression. *Cytokine*.

[42] Castley A., Williams L., James I., Guelfi G., Berry C., Nolan D. (2016). Plasma CXCL10, sCD163 and sCD14 levels have distinct associations with antiretroviral treatment and cardiovascular disease risk factors. *PLoS One*.

[43] Stiksrud B., Lorvik K. B., Kvale D. (2016). Plasma IP-10 is increased in immunological nonresponders and associated with activated regulatory T cells and persisting low CD4 counts. *Journal of Acquired Immune Deficiency Syndromes*.

[44] Platten M., Jung N., Trapp S. (2016). Cytokine and chemokine signature in *elite* versus *viremic controllers* infected with HIV. *AIDS Research and Human Retroviruses*.

[45] Ploquin M. J., Madec Y., Casrouge A. (2016). Elevated basal pre-infection CXCL10 in plasma and in the small intestine after infection are associated with more rapid HIV/SIV disease onset. *PLoS Pathogens*.

[46] Jacobs E. S., Keating S. M., Abdel-Mohsen M. (2017). Cytokines elevated in HIV elite controllers reduce HIV replication *in vitro* and modulate HIV restriction factor expression. *Journal of Virology*.

[47] Graham S. M., Rajwans N., Jaoko W. (2013). Endothelial activation biomarkers increase after HIV-1 acquisition: plasma vascular cell adhesion molecule-1 predicts disease progression. *AIDS*.

[48] Spertini O., Schleiffenbaum B., White-Owen C., Ruiz P., Tedder T. F. (1992). ELISA for quantitation of L-selectin shed from leukocytes in vivo. *Journal of Immunological Methods*.

[49] Matsumoto T., Miike T., Nelson R. P., Trudeau W. L., Lockey R. F., Yodoi J. (1993). Elevated serum levels of IL-8 in patients with HIV infection. *Clinical & Experimental Immunology*.

[50] Cloke T., Munder M., Bergin P. (2013). Phenotypic alteration of neutrophils in the blood of HIV seropositive patients. *PLoS One*.

[51] Browers N. L., Helton E. S., Huijbregts R. P., Groepfert P. A., Heath S. L., Hel Z. (2014). Immune suppression by neutrophils in HIV-1 infection: role of PD-L1/PD-1 pathway. *PLoS Pathogens*.

[52] Pawar R. S., Abhang S. A. (2017). Study of role of surfactant protein-D, malondialdehyde, protein carbonyl and its correlation with airflow obstruction (FEV 1% predicted) in patients with smoker chronic obstructive pulmonary disease (COPD). *Biochemistry & Analytical Biochemistry*.

[53] Plit M. L., Theron A. J., Fickl H., van Rensburg C. E. J., Pendel S., Anderson R. (1998). Influence of antimicrobial chemotherapy and smoking status on the plasma concentrations of vitamin C, vitamin E, *β*-carotene, acute phase reactants, iron and lipid peroxides in patients with pulmonary tuberculosis. *The International Journal of Tuberculosis and Lung Disease*.

[54] Waweru P., Anderson R., Steel H., Venter W. D. F., Murdoch D., Feldman C. (2013). The prevalence of smoking and the knowledge of smoking hazards and smoking cessation strategies among HIV- positive patients in Johannesburg, South Africa. *The South African Medical Journal*.

[55] Ohtsuki T., Satoh K., Omura J. (2017). Prognostic impacts of plasma levels of cyclophilin A in patients with coronary artery disease. *Arteriosclerosis, Thrombosis, and Vascular Biology*.

[56] Ande A., McArthur C., Ayuk L. (2015). Effect of mild-to-moderate smoking on viral load, cytokines, oxidative stress, and cytochrome P450 enzymes in HIV-infected individuals. *PLoS One*.

[57] Kucuk C., Sozuer E., Gursoy S. (2006). Treatment with Met-RANTES decreases bacterial translocation in experimental colitis. *The American Journal of Surgery*.

[58] Brozyna S., Ahern J., Hodge G. (2009). Chemotactic mediators of Th1 T-cell trafficking in smokers and COPD patients. *COPD*.

[59] Milot J., Meshi B., Rad M. T. S. (2007). The effect of smoking cessation and steroid treatment on emphysema in guinea pigs. *Respiratory Medicine*.

[60] Podolec J., Kopec G., Niewiara L. (2016). Chemokine RANTES is increased at early stages of coronary artery disease. *Journal of Physiology and Pharmacology*.

[61] Simeoni E., Winkelmann B. R., Hoffmann M. M. (2004). Association of RANTES G-403A gene polymorphism with increased risk of coronary arteriosclerosis. *European Heart Journal*.

[62] Braunersreuther A., Steffens S., Arnaud C. (2008). A novel RANTES antagonist prevents progression of established atherosclerotic lesions in mice. *Arteriosclerosis, Thrombosis and Vascular Biology*.

[63] Risso K., Guillouet-de-Salvador F., Valerio L. (2017). COPD in HIV-infected patients: CD4 cell count highly correlated. *PLoS One*.

[64] Satoh K., Fukumoto Y., Sugimura K. (2013). Plasma cyclophilin A is a novel biomarker for coronary artery disease. *Circulation Journal*.

[65] Zhang M., Tang J., Yin J. (2018). The clinical implication of serum cyclophilin A in patients with chronic obstructive pulmonary disease. *International Journal of Chronic Obstructive Pulmonary Disease*.

[66] Abd-El-Ghany M. M., Sarhan I. I., Shawky S. M., El-Okda E. E., Kamel C. R. (2015). Prevalence of environmental acquired cadmium nephropathy among smokers. *International Journal of Advanced Research in Biological Sciences*.

[67] Saddiwal R., Hebbale M., Sane V. D., Hiremutt D., Gupta R., Merchant Y. (2017). Estimation of serum beta 2-microglobulin levels in individuals exposed to carcinogens: clinical study in Indian population. *Journal of Maxillofacial and Oral Surgery*.

[68] Rist P. M., Jiménez M. C., Rexrode K. M. (2017). Prospective association between *β*_2_-microglobulin levels and ischemic stroke risk among women. *Neurology*.

[69] Kodidela S., Ranjit S., Sinha N., McArthur C., Kumar A., Kumar S. (2018). Cytokine profiling of exosomes derived from the plasma of HIV-infected alcohol drinkers and cigarette smokers. *PLoS One*.

[70] Levy J. A. (2009). The unexpected pleiotropic activities of RANTES. *The Journal of Immunology*.

[71] Ilumets H., Rytilä P., Demedts I. (2007). Matrix metalloproteinases -8, -9 and -12 in smokers and patients with stage 0 COPD. *International Journal of Chronic Obstructive Pulmonary Disease*.

[72] Louhelainen N., Stark H., Mazur W., Rytilä P., Djukanovic R., Kinnula V. L. (2010). Elevation of sputum matrix metalloproteinase-9 persists up to 6 months after smoking cessation: a research study. *BMC Pulmonary Medicine*.

[73] Snitker S., Xie K., Ryan K. A. (2013). Correlation of circulating MMP-9 with white blood cell count in humans: effect of smoking. *PLoS One*.

[74] Bchir S., ben Nasr H., Bouchet S. (2017). Concomitant elevations of MMP-9, NGAL, proMMP-9/NGAL and neutrophil elastase in serum of smokers with chronic obstructive pulmonary disease. *Journal of Cellular and Molecular Medicine*.

